# Supplementary material for: Ordering sequential competitions to reduce order relevance: Soccer penalty shootouts
Source: PLoS One. 2020 Dec 30;15(12):e0243786. doi: 10.1371/journal.pone.0243786 (PMC7773190; doi:10.1371/journal.pone.0243786)
Supplement: S2 File — (PDF) [file pone.0243786.s002.pdf]

1 Data changes since initial submission

This is a description of the changes made to the data after the initial submission to PLOS one. The data set used in the initial submission had 1635 shootouts. The changes we made resulted in 15 shootouts being affected. This includes 12 shootouts that were dropped and three shootouts that were corrected.

The changes can be classified into 2 groups:

1.1 Comparison with data from past literature

When we matched our data to that used in previous literature, we found that the shooting sequence did not match for 11 shootouts. For many of these games, data reported on different websites was contradictory. In order to resolve these differences, we looked for video footage of each of these shootouts. We made the following changes after this:

1. Our data was correct for four of these shootouts, so we left these shootouts in our data as is.

2. Our data was incorrect for three of the shootouts but we have now corrected them (Table 1.2 in excel file).

3. For the remaining four conflicting shootouts, we could not find any video footage. Therefore, we decided to exclude these shootouts from our data (Table 1.2)

1.2 Shootouts reported twice

In the data that was collected manually (for the Spanish league), we found that eight shootouts had been reported twice from different sources, either because of difference in dates, differences in team names or the order in which teams were assigned to being home and away. These games are mentioned in Table 1.2 and the duplicates have been dropped from our revised data.

Competition	Date	Home Team	Away Team	Starting team Won
Gold Cup	1/27/2002	Mexico	South Korea	0
DFB-Pokal	2/6/1985	FC Saarbrücken	VfB Stuttgart	0
EURO	6/21/1980	CSSR	Italy	0

Table 1: Corrected shootouts

Competition	Date	Home Team	Away Team	Starting team Won
Africa Cup	2/12/2000	South Africa	Tunisia	1
League Cup	11/27/2001	Bolton Wanderers	Southampton FC	1
Gold Cup	1/26/2002	Canada	Martinique	1
DFB-Pokal	8/30/2002	SpVgg Unterhaching	FSV Mainz 05	0

Table 2: Shootouts with ambiguous data: Now dropped

Competition	Date	Home Team	Away Team	Starting team Won
Copa del Rey	12/28/1977	RC Deportivo de La Coruña	RCD Español	1
Copa del Rey	2/2/1977	Burgos CF	UE Lleida	1
Copa del Rey	1/22/1992	CA Osasuna [Pamplona]	RC Deportivo de La Coruña	1
Copa del Rey	1/22/1992	Valencia CF	FC Barcelona	0
Copa del Rey	2/22/1989	RC Celta	CA Osasuna	0
Copa del Rey	1/24/1979	Atletico de Madrid	Real Madrid	1
Copa del Rey	10/27/1976	Linares CF	Real Racing Club de Santander	1
Copa del Rey	9/17/1986	Villareal	Valencia	1

Table 3: Repeated shootouts: Now dropped

2 Shootouts with missing or no sequence

In addition to the data that has been used in the paper, we found 894 games where our data source identified that a shootout had taken place. However, there was either no data on the shooting order or the order specified was invalid i.e. could not have occurred based on the rules of the game (eg: the shooting order reports 4 shots taken by each team even though the score was 3-0 at the end of the third round). The file *shootouts_invalid_no_sequence.csv* lists these games.
