# Supplementary material for: Ordering sequential competitions to reduce order relevance: Soccer penalty shootouts
Source: PLoS One. 2020 Dec 30;15(12):e0243786. doi: 10.1371/journal.pone.0243786 (PMC7773190; doi:10.1371/journal.pone.0243786)
Supplement: Attachment — Submitted filename: responseR1_submission.pdf [file pone.0243786.s004.pdf]

Summary of changes:

We are thankful for the reviewers and the editor for the constructive comments in their review. We apologize for the delay in submitting the revised manuscript (which in part was due to the pandemic). We have worked hard to address all of the comments, which involved some additional data analysis and the relevant literature. Specifically:

- We conducted some data analysis to test for the markovian assumption of our model.
- Discuss the implications of this assumption and the psychological mechanisms that are been ruled out.
- Provide some discussion and analysis explaining why Thue-Morse sequence is not the most equitable sequence when applied to penalty shootouts.
- Worked to make the data openly available, as well as all the programming codes used to make the calculations and figures in the paper.

We provided detailed reponses to each of the reviewers comments below (the reviewers original comment is formatted as a quotation, followed by our response in regular formatting).

Reviewer #1:

> Summary:
> This work compiles an extensive dataset of soccer penalty shootouts and conduct a counterfactual analysis in order to inform the decision by a policy unit about whether or not to alternate order of the team shooting first in each round of penalty shootouts.
> The paper reviews a brief literature suggesting mixed conclusions about whether or not there is an advantage for first shooters in penalty shooters. The first shooter being drawn by a fair coin, such advantage increases the noise-to-talent ratio in what determines outcomes of soccer competitions. The paper starts by compiling a large dataset of games and confirms a statistically significant advantage of the first shooter.
> Because penalty shootouts do not occur so frequently, conducting a randomized experiments powered to see if the first shooter advantage is removed by alternating the order of who shoots first across rounds would take too long. The paper takes another approach. The work estimates transition probabilities from states to states, where a state is defined by the score difference and the stage in the shootout. Under the assumption that scoring probability of each team is only influenced by these two variables at each point in time, the authors are able to simulate what would outcomes be under alternate shooting

> orders that are being considered by a policymaker. They find that the alternating orders would reduce the first-shooter advantage.
>
> (3) Experiments, statistics, analyses are high-standard and described in detail: Yes, the model used is described in details and clearly. Confidence intervals are generated using a bootstrap procedure that is properly described.
> Introductory discussion notes that there is no difference in the first mover advantage across competitions. Rather, each competition has a relatively small sample and thus power to detect if first shooter advantage differs across them is insufficient to make the conclusion that is does not differ. This should be rephrased but is not central for the core of the analysis.

Response: We agree that the comparison across competitions has low statistical power due to the small sample for each competition. As suggested, we have rephrased this as follows:

The resulting p-value of this test is 0.72, suggesting there are no systematic differences in the first-mover advantage across competitions, although the statistical power of this test is low due to the relatively small sample size for each competition.

> (4) Conclusions supported by the data: Partially. The core assumptions for validity of the counterfactual are clearly stated but should be discussed further. In particular, no-path-dependence is a strong assumption that may or may not be reasonable. If Team A is leading 2-1 at the beginning of the third shootout, there is a good chance that psychological effects leading to advantages depend on whether Team B missed on the first or the second shot. The authors could discuss why they believe this assumption is reasonable and provide empirical support for this assumption using the data.

Response: First, we elaborate a bit on why we make this assumption. There are in essence two main ways we see to address the challenge of estimating the effect of alternative sequences using existing data. One would be to model the psychological mechanisms that influence scoring probabilities in penalty shootouts. Such models would necessarily be simplified and reflect only a subset of potential mechanisms that may coexist. Our approach is to use a probability network, which does not rely on the underlying psychological mechanisms but instead makes a markovian assumption. A second advantage of this assumption is that in our experience, practitioners seems to be able to understand it and has expressed trust in the resulting analysis capturing the main drivers of the advantage of the starting team in penalty shootouts. While the markovian assumption is quite standard in modeling sequential stochastic systems, it does not in any way guarantee that it is a good assumption in the setting of soccer penalty shootouts. In the spirit of robustness and trying to shoot down our results to see if there may be alternative explanations of the results obtained, we

had already done some analysis to challenge this assumption before submitting the first version. In an effort to keep the paper short and easily accessible, we did not include this analysis in the first submission. We will here give some analysis made in this respect.

The most general way to account for the history of the shootout is to consider the result of every preceding shot. However, it is reasonable to expect that the outcomes of the most recent shots will have a larger effect than those of earlier shots. We developed an econometric model to test this assumption.

Recall that we model the evolution of the penalty shootout through a network model where the state captures the goal difference in the shootout. The state evolves from one round to the next by increasing, decreasing or maintaining the goal difference. Consequently, we model the state transition probabilities using an ordered probit model with three possible outcomes, including covariates that capture the states in the preceding rounds. Specially, the latent variable of the ordered probit us specified as:

$$y_{\mathrm{rsm}} = \alpha_{\mathrm{rs}} + \gamma I_m^{\mathrm{APH}} + \beta_1 I^+_{(r-1)m} + \beta_2 I^-_{(r-1)m} + \epsilon_{\mathrm{rsm}}$$

where:

$\underline{\mathrm{y_{rsm}}}$ is the outcome of round $\underline{\mathrm{r}}$ in match $\underline{\mathrm{m}}$, when the score difference is $y_{rsm} \in \{1, -1, 0\}$

α_{rs} is the fixed effect for round r when the score difference is s, i.e. a fixed effect for every state.

I^m_{APH} is an indicator for whether this shootout belongs to an APH competition.

$I^+_{(r-1)m}$ is an indicator variable for whether the previous round increased the goal difference in favor of team A.

$I^-_{(r-1)m}$ is an indicator variable for whether the previous round decreased the goal difference in favor of team A.

For states $(r, s) \in \{(3, \pm 2), (4, \pm 2), (5, \pm 1)\}$ and all states in the first two rounds, the path to the state is unique, and hence we exclude these states from the analysis.

The covariates $I^+_{(r-1),m}$ and $I^-_{(r-1),m}$ capture state dependence. Therefore, $\beta_1 = \beta_2 = 0$ would indicate that the path in which the state was reached does not affect the transition probabilities to the next state, providing support for the Markovian assumption. We use a likelihood ratio test, setting the Null to the restricted model $\beta_1 = \beta_2 = 0$. We also considered a similar model using two preceding rounds. The following table shows the p-values of the LR tests along with the corresponding log-likelihoods of all three models.

	Base	Preceding round	Two preceding rounds
Log Likelihood	-4231.05	-4230.422	-4228.2
LR test p-value		0.5335	0.6885

Therefore, we do not find evidence that the outcomes of the preceding rounds have a significant impact on the outcome of the current round (conditioning on the state of the shootout). This providing support for the Markov assumption. We provide a short description of this test in the paper at the end of Section "A network model of penalty shootouts".

> (5) Presented in intelligible fashion: Yes but this can be improved. Overall the key approach and core results are very well presented. The secondary analysis of the network, from lines 176 to lines 205 would benefit from more structure. What are the main takeaways from these various tests? Currently this part of the paper does not seem very useful because its takeaways are not explicitly connected to the key conclusions of the paper.

Response: Revising the submission, we realized that the previous version included by mistake a wrong version of Figure 1 (which is submitted separately from the main manuscript). The text in the paper was inconsistent with the figure, which may in part explain the confusion. We apologize for the confusion created and hope that you will find the discussions related to the network and its results to be integrated with the text in a more comprehensive way.

This part of the paper makes two main contributions:(i) provides further evidence on the advantage of the first-shooter in penalty shootouts; (ii) develops the structured network model which is necessary to analyze alternative sequencing of the penalty shootout. Although the first contribution is a stepping stone for the second one, we find the network model and the counterfactuals that we analyze based on it to be the main contribution of the paper. Following your suggestion, we have worked on the writing and structuring of the analysis presented in section "A network model of penalty shootout", to explain the value of the network model to analyze the first-shooter advantage and the purpose of the tests that are conducted, before jumping into the technical details of the tests. Thank you for the suggestion.

> (7) Data availability: Authors should explain in greater details what makes them unable to provide the dataset being used. Many of the data sources being used have no restrictions (such as wikipedia) and the authors could at least provide a compiled dataset with this data, leaving out the data from sources that did not agree to share data for publication.

Response: Thanks for the suggestion. Indeed, all the data used in this study is from public sources, and so we decided to make our dataset openly available

upon publication for replication purposes. The data will be available in the following repository:

https://datos.uchile.cl/dataset.xhtml?persistentId=doi:10.34691/FK2/QEZXKG

In addition, we revised the data in detail to correct for possible inconsistencies. First, we compared our data with datasets on penalty shootouts that have been used in the literature. We found some inconsistencies in the data for 11 shootouts, so we revised video footage on each of these shootouts to correct the data. Four of these shootouts were correctly coded in our dataset, and three of them were not (and were corrected). We couldn't find video footage on the remaining 4 shootouts, so we opted to exclude these from the analysis. Second, we identified some duplicates in the data because they were reported with different dates or team names in the source. We dropped three duplicate penalty shootouts. These data corrections are described in detail in the supplemental document provided in revision.

> Conclusion
> The paper makes a very clear demonstration of its approach to estimating effects of a counterfactual order in penalty shootouts. A major assumption (no path dependence in transition probabilities) is clearly acknowledged but could be a little more discussed.

Response: Thank you for the useful suggestions. We now provide formal tests of the no path dependence assumption (see our response to point (4)). In addition, as suggested by another reviewer, we discussed alternative explanations that are been ruled out by this assumption.

> The paper would also gain in interest if it could tie some of its conclusions to the cited literature on the psychology of penalty shootouts. Are there psychological reasons to explain that the Thue-Morse order reverses the Team A advantage? Are the intermediate findings from the network (currently a little disconnected from the rest of the paper), such as the fact that Team A's advantage starts after the 2nd round, naturally related to any psychological mechanism discussed in this literature?

Response:

When first doing this analysis, we were also a bit surprised to why ABBA (which is T_2, Thue-Morse of degree 2) resulted in better evening out the difference in win probabilities than the full Thue-Morse sequence T_n. The initial surprise was based on Thue-Morse being considered the "gold standard" in fair sequential division ([2],[4]). While there may also be particular psychological mechanisms that could shed light on this, it appears that a lot can be explained by type

of problems for which the Thue-Morse sequence have been reported to yield superior results: Problems where the structure and parameters are stationary. For soccer penalty shootouts, there are two key deviations from this: The first is that the round-wise performance advantage of the first-shooting team, where the first two rounds does not have significant advantage of the first-shooting team, and after the second round the advantage of the first shooting team may differ between rounds. Second, the structure itself is not stationary, but rather start with best-out-of-5 shots, and if a winner is still not determined turns to repetitions of best-out-of-1 shot.

To explore this, we considered the following variations of our analysis:

Case 1 The model considered in our paper, with all probabilities being state-dependent (round and score) and starting with best-out-of-5 shots, then best-out-of-1 shot.

Case 2 Stationary structure with best-out-of-1 shot from the start, and with non-stationary probabilities, where for each round the trinomial transition probabilities are estimated by weighted averages for that round.

Case 3 First best-out-of-5 shots, then best-out-of-1 shot, using the same trinomial transition throughout which is estimated by weighted averages of all transition probabilities.

Case 4 Stationary structure with best-out-of-1 shot from the start, and with stationary probabilities, where the same trinomial transition probabilities are used throughout and estimated by weighted averages of all transition probabilities.

So Case 1 is the model of our paper, Case 2 considers stationary structure, Case 3 considers stationary transition probabilities and Case 4 considers both stationary structure and stationary probabilities.

The resulting win probabilities of team A are given in the following table (for each case, the sequence with win probability closest to 0.5 is in bold):

Case	ABAB	ABBA	T-M
1	0.5458	**0.5118**	0.4875
2	0.5199	**0.5027**	0.4865
3	0.5412	**0.5095**	0.4814
4	0.5306	0.5073	**0.5023**

We see that for Case 4, where both the structure and probabilities are stationary, the Thue-Morse sequence will result in the win probabilities of the two teams being the closest to 0.5, and for the other 3 cases, the ABBA sequence achieves the probabilities closest to 0.5. This suggest that for soccer penalty shootouts, it happens to be that the ABBA sequence evens the probabilities better than the Thue-Morse sequence, and that this can in part be explained by the non-stationary structure and probabilities. Following the reviewer's suggestion, we briefly discuss this issue at the end of section "Alternative orders".

Reviewer #2:

> This paper provides evidence of the importance of order in sequential competitions in the context of penalty shootouts. The authors contribute to the current literature by compiling a comprehensive dataset of penalty shootouts, providing new estimates of the first mover advantage and constructing counterfactuals for different order sequences using a probability network model. The evidence presented is concise and compelling, the analysis of the paper is appropriate (even though the assumptions are strong) and the paper is clear and well written. The following comments may improve the presentation of the paper.

> 1. First, the dataset the authors compile might be of great interest to other researchers in the field, so, in the spirit of the journal, I encourage the authors to make the data publicly available. In the case that it is not possible to do so; it would be useful to provide more complete descriptive statistics. In particular, I would have found it useful to breakdown the % of Team A winning statistic by pre and post 2003 (given that the rules change), so that the numbers can be compared more directly with the literature.

Response: Thanks for the suggestion. Indeed, all the data used in this study is from public sources, and so we decided to make our dataset openly available upon publication for replication purposes. The data will be available in the following repository:

https://datos.uchile.cl/dataset.xhtml?persistentId=doi:10.34691/FK2/QEZXKG

In addition, we revised the data in detail to correct for possible inconsistencies. First, we compared our data with datasets on penalty shootouts that have been used in the literature. We found some inconsistencies in the data for 11 shootouts, so we revised video footage on each of these shootouts to correct the data. Four of these shootouts were correctly coded in our dataset, and three of them were not (and were corrected). We couldn't find video footage on the remaining 4 shootouts, so we opted to exclude these from the analysis. Second, we identified some duplicates in the data because they were reported with different dates or team names in the source. We dropped three duplicate penalty shootouts. These data corrections are described in detail in the supplemental document provided in revision.

> 2. Given that the IFAB decided not to change the system it might be worth it discussing a bit more in detail what costs are associated with changing the shootout orders. This might help inform the validity of the modelling assumptions and put the results in context. For

instance, there might be a strategic reasoning to choosing which players shoot first or last that changes with the shootout order. It would also be useful to mention, briefly, what were the results of FIFA's test for U17 and U20 tournaments.

Response:

It is not easy to capture such costs of changing the sequence of the penalty shootout, but it can be useful to categorize them. There would be a fixed cost in terms of implementation and training all stakeholders (including the millions of fans). Variable costs would include extra complexity and confusion that would remain after the implementation phase. In casual conversations with our main contacts in FIFA, the tests that were performed were described as causing massive confusion. Although we do not have formal information about it, our impression was that this was the main reason why the experiments has been stopped for now. In addition to the cost implications, the number of changes FIFA/IFAB can do to the laws of the game is in practice constrained.

In terms of deciding the order of shooters for a team, the first part of this decision is when to make it. This could be (a) before the match, (b) as it becomes clear that there will be a shootout (the score will be tied after extra time), (c) between the decision of which team goes first and the start of the shootout, and (d) during the shootout. Second, who will make the decision. While we are not aware of archival data about this, it is a topic that we have discussed with contacts in professional soccer (including with the brother of one of the authors who played professionally for 17 years, including English Premier League, Italian Serie A and 46 games for his countries national team. He also was the head opponent analyst for a national team for the qualification and during the World Cup 2018, for which this team was in a penalty shootout. The general opinion seems to be that it is the head coach who is the principal decision maker and that the decision timings (c) and (d) are unlikely. Several expressed that in their experience, a hybrid of (a) and (b) is common, where there is a base selection/sequence prior to the match, and this one may be adjusted at time (b) based on shooter availability (some likely shooters may have been substituted) and how the players feel (experiencing cramps after 120 minutes of play is not the ideal physical condition for shooting a penalty). Also, there is limited possibilities for communication between the coaching staff and players between the coin toss and the start of the shootout, both in terms of time, distance and noise level. This anecdotal evidence suggest that it is unlikely that the differences observed between the shootout performance between teams A and B would be a result of scheduling decisions to cater to it.

3. In the discussion of the mixed evidence for first mover advantage in the literature review I would also mention that most players perceive shooting first as being better, as suggested by survey evidence in Apesteguia and Palacios-Huerta 2010.

Response: Thank you for the suggestion. We now include this result from

Apesteguia and Palacios-Huerta 2010 in the introduction of the paper.

> 4. One of the new results of the network model with round dependent states is that the first mover advantage becomes statistically significant after round 2 for tied states. It is not immediately intuitive why this would be the case. Is there a mechanism that could explain it? For example, if trainers placed their strongest players (least likely to be influenced by behavioral aspects) in rounds 1,2 and the last round, then maybe we start seeing the effect in round 3 because the players are weaker.

Response: Thanks for the suggestion. A first thing to note that, if shooting performance was not affected by sequencing, then there would be no advantage of reordering players based on their performance. To see this, for every possible realization of the shootout were a team loses, there is not reordering of the shots of the first 5 rounds that would change the outcome. Hence, it is actually the impact of sequence on performance that may motivate a team to strategize which players go first. Moreover, it is not evident what is the optimal strategy when players are subject to psychological pressure. On one side, it is better to put the best shooters first to gain advantage and put pressure on the opposing team. However, it is also beneficial to leave the players that are more resilient to pressure to shoot later in the shootout. This involves a complex dynamic optimization problem, which is an interesting avenue of future research motivated by this work.

To further explore this issue, we conducted some descriptive analysis to study which players are more likely to shoot first. First, we need a measure of the performance of players in shooting penalties, which is not directly available in our data. As a proxy, we identified players who scored a penalty during the regular time of the games in our sample. We found 311 scored in-game penalties, and 80% of these players took a shot in the shootout (compared to 48% of a random player in our sample). Notice that some of the players in the 20% that do not participate in the penalty shootout were substituted before the end of the game. This result suggest that, as expected, experienced penalty shooters are more likely to take a shot in the penalty shootout. We then analyze in which round did players who scored an in-game penalty took their penalty shot in the penalty shootout; Figure compares the shooting round between these players (n=252) and all other players (n=17082). More than 40% of the in-play penalty scorers took their shot in the first round of the penalty shootout, compare to 18% for the other players. Indeed, there is evidence that this apparently more experienced players shot first in the shootout. The scoring probability of these experienced shooters is 68% (CI = [62.2,73.8]) compared to the 73% of the average scoring rate; there is not enough statistical power to determine if the performance of this players was different from the rest of the population. Altogether, this analysis provides some suggestive evidence that players with more experience in shooting penalties tend to shoot in the initial rounds of a shootout, which may in part explain why the sequencing does not have a significant effect in the first two

rounds. Given the small sample used in this analysis, we decided not to include it in the paper. Nevertheless, it provides an interesting question for future research, which would require more detailed data on players' experience shooting penalties.
.

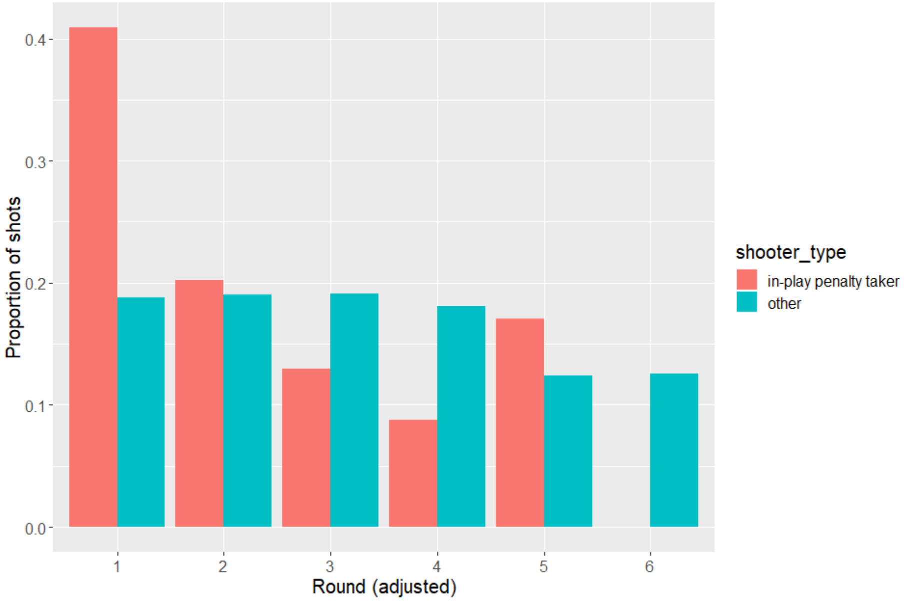

Figure 1: Comparison of shooting rounds between players that scored an in-play penalty and all other players.

5. The assumption that transition probabilities depend only on the state and round number is very strong. Together with the assumption that the teams are identical this makes the counterfactual analysis highly stylized and potentially misleading as the transition probabilities computed from the data are likely to come from path dependent process and strategic considerations when changing the order might be important. As the authors point out a more in-depth analysis would be appropriate for a longer paper. However, to make the results more robust, I would add a brief discussion of what mechanisms are ruled out by the assumption (and the "mirror image" trick) and what direction this is likely to bias the results towards (my prior is that there is a downward bias when using the mirror trick). This will help contextualize why the T-M order reverts the advantage (surprising given that it is supposed to be 'fair'), and might advocate further for not changing the order to ABBA.

Response: The comments has two parts: (i) further discussion of the validity

of no path dependence; (ii) discuss alternative explanations that are been ruled out by this assumption.

In terms of the first part, we conducted some additional statistical analysis to further support this assumption. Recall that we model the evolution of the penalty shootout through a network model where the state captures the goal difference in the shootout. The state evolves from one round to the next by increasing, decreasing or maintaining the goal difference. Consequently, we model the state transition probabilities using an ordered probit model with three possible outcomes, including covariates that capture the states in the preceding rounds. Specially, the latent variable of the ordered probit us specified as:

$$y_{\text{rsm}} = \alpha_{\text{rs}} + \gamma I_m^{\text{APH}} + \beta_1 I^+_{(r-1)m} + \beta_2 I^-_{(r-1)m} + \epsilon_{\text{rsm}}$$

where:

$\underline{\text{y}_{\text{rsm}}}$ is the outcome of round $\underline{\text{r}}$ in match $\underline{\text{m}}$, when the score difference is $y_{rsm} \in \{1, -1, 0\}$

α_{rs} is the fixed effect for round r when the score difference is s, i.e. a fixed effect for every state.

I^m_{APH} is an indicator for whether this shootout belongs to an APH competition.

$I^+_{(r-1)m}$ is an indicator variable for whether the previous round increased the goal difference in favor of team A.

$I^-_{(r-1)m}$ is an indicator variable for whether the previous round decreased the goal difference in favor of team A.

For states $(r, s) \in \{(3, \pm 2), (4, \pm 2), (5, \pm 1)\}$ and all states in the first two rounds, the path to the state is unique, and hence we exclude these states from the analysis.

The covariates $I^+_{(r-1),m}$ and $I^-_{(r-1),m}$ capture state dependence. Therefore, $\beta_1 = \beta_2 = 0$ would indicate that the path in which the state was reached does not affect the transition probabilities to the next state, providing support for the Markovian assumption. We use a likelihood ratio test, setting the Null to the restricted model $\beta_1 = \beta_2 = 0$. We also considered a similar model using two preceding rounds. The following table shows the p-values of the LR tests along with the corresponding log-likelihoods of all three models.

	Base	Preceding round	Two preceding rounds
Log Likelihood	-4231.05	-4230.422	-4228.2
LR test p-value		0.5335	0.6885

Therefore, we do not find evidence that the outcomes of the preceding rounds

have a significant impact on the outcome of the current round (conditioning on the state of the shootout). This providing support for the Markov assumption. We provide a short description of this test in the paper at the end of Section “A network model of penalty shootouts".

On the second part, the assumption of no-path dependence rules out some mechanisms that have been reported in the literature. [5], [1] and [6] provide evidence that after receiving penalty shots repeatedly in one direction, goalkeepers are more likely to dive in the opposite direction, an example of the Gambler’s fallacy [7]; although [3] later discussed that penalty shootout may not be well-suited to analyze this effect. This assumption also rules out "hot-hand" phenomena, in which a shooting team or the keeper may exhibit streaks of success / failures across rounds. This phenomena has been documented for free-throws in basketball by [8], but to our knowledge has not been reported for penalty shootouts.

We have added this discussion in the paper right before Figure 1.

References

[1] Simcha Avugos, Ofer H Azar, Nadav Gavish, Eran Sher, Michael Bar-Eli, et al. Goal center width, how to count sequences, and the gambler’s fallacy in soccer penalty shootouts. Judgment and Decision Making, 14(1):98–108, 2019.

[2] Steven J Brams and Alan D Taylor. Fair Division: From cake-cutting to dispute resolution. Cambridge University Press, 1996.

[3] Sebastian Braun and Ulrich Schmidt. The gambler’s fallacy in penalty shootouts. Current Biology, 25(14):R597–R598, 2015.

[4] Joshua Cooper and Aaron Dutle. Greedy galois games. The American Mathematical Monthly, 120(5):441–451, 2013.

[5] Erman Misirlisoy and Patrick Haggard. Asymmetric predictability and cognitive competition in football penalty shootouts. Current Biology, 24(16):1918–1922, 2014.

[6] Abhishek Sarkar. The Gambler’s Fallacy and Hot Outcome: Cognitive Biases or Adaptive Thinking for Goalkeepers’ Decisions on Dive Direction During Penalty Shootouts. PhD thesis, Bowling Green State University, 2017.

[7] Amos Tversky and Daniel Kahneman. Belief in the law of small numbers. Psychological bulletin, 76(2):105, 1971.

[8] Gur Yaari and Shmuel Eisenmann. The hot (invisible?) hand: can time sequence patterns of success/failure in sports be modeled as repeated random independent trials? PloS one, 6(10):e24532, 2011.
